# Supplementary material for: Foldamers reveal and validate therapeutic targets associated with toxic α-synuclein self-assembly
Source: Nat Commun. 2022 Apr 27;13:2273. doi: 10.1038/s41467-022-29724-4 (PMC9046208; doi:10.1038/s41467-022-29724-4)
Supplement: Supplementary file 3 — Description of Additional Supplementary Files [file 41467_2022_29724_MOESM3_ESM.pdf]

Title: Supplementary Movie 1:
Description: A representative 3D movie (from a series of Z-stack images) of an NL5901 worm untreated at day 8 of adulthood.

Title: Supplementary Movie 2:
Description: A representative 3D movie (from a series of Z-stack images) of an NL5901 worm at day 8 of adulthood after treating with SK-129
