# Supplementary material for: Foldamers reveal and validate therapeutic targets associated with toxic α-synuclein self-assembly
Source: Nat Commun. 2022 Apr 27;13:2273. doi: 10.1038/s41467-022-29724-4 (PMC9046208; doi:10.1038/s41467-022-29724-4)
Supplement: Supplementary file 6 — Reporting Summary [file 41467_2022_29724_MOESM6_ESM.pdf]

nature portfolio

Corresponding author(s): Sunil Kumar

Last updated by author(s): Feb 28, 2022

Reporting Summary

Nature Portfolio wishes to improve the reproducibility of the work that we publish. This form provides structure for consistency and transparency in reporting. For further information on Nature Portfolio policies, see our Editorial Policies and the Editorial Policy Checklist.

Statistics

For all statistical analyses, confirm that the following items are present in the figure legend, table legend, main text, or Methods section.

n/a	Confirmed	
☐	☒	The exact sample size (n) for each experimental group/condition, given as a discrete number and unit of measurement
☐	☒	A statement on whether measurements were taken from distinct samples or whether the same sample was measured repeatedly
☐	☒	The statistical test(s) used AND whether they are one- or two-sided<br>*Only common tests should be described solely by name; describe more complex techniques in the Methods section.*
☒	☐	A description of all covariates tested
☒	☐	A description of any assumptions or corrections, such as tests of normality and adjustment for multiple comparisons
☐	☒	A full description of the statistical parameters including central tendency (e.g. means) or other basic estimates (e.g. regression coefficient) AND variation (e.g. standard deviation) or associated estimates of uncertainty (e.g. confidence intervals)
☐	☒	For null hypothesis testing, the test statistic (e.g. F, t, r) with confidence intervals, effect sizes, degrees of freedom and P value noted<br>*Give P values as exact values whenever suitable.*
☒	☐	For Bayesian analysis, information on the choice of priors and Markov chain Monte Carlo settings
☒	☐	For hierarchical and complex designs, identification of the appropriate level for tests and full reporting of outcomes
☒	☐	Estimates of effect sizes (e.g. Cohen's d, Pearson's r), indicating how they were calculated

Our web collection on statistics for biologists contains articles on many of the points above.

Software and code

Policy information about availability of computer code

Data collection	The software programs and the instrument used for data collection in this manuscript (open source and commercial) are clearly indicated in the Methods section in the subheadings for various methods and techniques. The processing of the confocal images of HEK cells, C elegans, and neuronal cells was carried out using ImageJ software from Olympus. The NMR data collected using MestReNova NMR (Version 12.0.4) software and Origin 2020b.
Data analysis	The data analysis for CD, ThT, Fluorescence polarization titrations, MTT and LDH toxicity assays, Inclusion counts for HEK cells and C elegans, Activity counts for C elegans, were carried out using OriginPro software (Version 9.1), Origin 2020b, and GraphPad Prism (Version 9.3.1) softwares. The NMR data analysis was carried out using MestReNova NMR (Version 12.0.4) software and Origin 2020b. The analysis of the confocal images of HEK cells, C elegans, and neuronal cells was carried out using ImageJ software from Olympus. In the flow cytometry experiments, the intracellular aggregation in HEK cells was analyzed and plotted using Cell Sorter Software (Version 1.7, LE-SH800 Series, Sony, San Jose, CA).

For manuscripts utilizing custom algorithms or software that are central to the research but not yet described in published literature, software must be made available to editors and reviewers. We strongly encourage code deposition in a community repository (e.g. GitHub). See the Nature Portfolio guidelines for submitting code & software for further information.

Data

Policy information about availability of data

All manuscripts must include a data availability statement. This statement should provide the following information, where applicable:

- Accession codes, unique identifiers, or web links for publicly available datasets
- A description of any restrictions on data availability
- For clinical datasets or third party data, please ensure that the statement adheres to our policy

All the datasets for experiments in the manuscript and supplementary information will be available in the 'Source Data File'. The data availability statement has been included in the main manuscript as well and it now reads as : 'All the datasets generated and analyzed during the current study are also available from the corresponding author. Source data are provided with this paper. '

Field-specific reporting

Please select the one below that is the best fit for your research. If you are not sure, read the appropriate sections before making your selection.

- [x] Life sciences
- [ ] Behavioural & social sciences
- [ ] Ecological, evolutionary & environmental sciences

For a reference copy of the document with all sections, see nature.com/documents/nr-reporting-summary-flat.pdf

Life sciences study design

All studies must disclose on these points even when the disclosure is negative.

Sample size	The in vitro experiments were carried out with three independent experiments (n = 3) and each independent experiments was repeated four times. For NMR experiments n=1, but at least one NMR experiment was repeated to confirm the reproducibility (data was not shown in the manuscript). Also, the NMR experiments between alpha-synuclein and SK-129 were conducted at different stoichiometric ratios and a consistent trend of the change in the chemical shift volumes was observed as a function of stoichiometric ratios, which supports the reproducibility of the data. Similarly, all CD experiments were conducted one time and the CD experiments between αS and SK-129 (or LUVs, DOPS) were conducted at different stoichiometric ratios and a consistent trend of the change in the CD signals was observed as a function of stoichiometric ratios (of αS and SK-129/DOPS, LUVs), which supports the reproducibility of the data.<br>The cellular experiments (Proteostat, cell viability etc) were carried out using four independent experiments (n = 4) and each experiment consisted of four technical replicates. For the in vivo experiments (C elegans), the experiments were carried out using at least three independent experiments (n = 3).
Data exclusions	No data was excluded from the manuscript.
Replication	All attempts for the replication of data were successful. The biophysical, cellular, and C elegans based experiments were replicated at least twice with a different batch of expressed proteins, new cultured cells, or freshly bleached c elegans strains, respectively.
Randomization	At least one randomization experiment was carried out to monitor the effect of molecules on synuclein aggregation in HEK cells, neuronal cells, and C elegans. The allocation of the samples was carried out randomly without any prior selection. The randomly allocated samples then treated with different experiment conditions to pursue the experiments.
Blinding	At least one blinding experiment was carried out to monitor the effect of molecules on alpha-synuclein aggregation in HEK cells, neuronal cells, and C elegans. During the blinding experiment, a second member of the lab pursued the blinding part of the experiments and further pursued the experiment.

Reporting for specific materials, systems and methods

We require information from authors about some types of materials, experimental systems and methods used in many studies. Here, indicate whether each material, system or method listed is relevant to your study. If you are not sure if a list item applies to your research, read the appropriate section before selecting a response.

Materials & experimental systems

n/a	Involved in the study	
[ ]	[x]	Antibodies
[ ]	[x]	Eukaryotic cell lines
[x]	[ ]	Palaeontology and archaeology
[ ]	[x]	Animals and other organisms
[x]	[ ]	Human research participants
[x]	[ ]	Clinical data
[x]	[ ]	Dual use research of concern

Methods

n/a	Involved in the study	
[x]	[ ]	ChIP-seq
[ ]	[x]	Flow cytometry
[x]	[ ]	MRI-based neuroimaging

Antibodies

Antibodies used	Anti-pS129-a-syn/ 81a (monoclonal) Catalog # 825702<br>Anti-p62 (monoclonal) Catalog # P0067<br>Anti-ubiquitin (monoclonal) Catalog # sc-8017 AF790<br>Anti- Tom 20 (monoclonal) Catalog # sc-17764 FITC<br>Goat anti-mouse Alexa Fluor 680 Catalog # A21058<br>Donkey anti-rabbit Alexa Fluor 568 Catalog # A10042<br><br>We have specified the dilution used for secondary antibodies in the methods section of the manuscript.<br>The dilution factor for both the primary and secondary antibodies were 1:1000 (v/v) in TBST buffer containing 5% BSA
Validation	All antibodies (Primary and secondary) are commercially available and are well established. All of the antibodies have been tested, validated, and utilized in multiple study that are published and well documented.

Eukaryotic cell lines

Policy information about cell lines

Cell line source(s)	HEK293 cells with transfection and over expression of WT-alpha-synuclein-YFP and A53T mutant alpha-synuclein-YFP were acquired via a generous Gift from Prof. Marc Diamond's lab (University of Texas Southwestern, Dallas, Texas) using a Materials Transfer Agreement. The SHSY 5Y cells were purchased from ATCC (product number: CRL-2266).
Authentication	HEK293 cells (Overexpressing WT-alpha-synuclein-YFP and A53T mutant alpha-synuclein-YFP) were authenticated by Prof. Marc Diamond's lab (University of Texas Southwestern, Dallas, Texas) and the SHSY 5Y cells were authenticated by ATCC (product number: CRL-2266). Our lab did not authenticate the cell lines.
Mycoplasma contamination	The testing of the contamination of cell lines were carried out by ATCC (for SHSY 5Y) or by Prof. Marc Diamond's lab (University of Texas Southwestern, Dallas, Texas) (HEK293 modified cell lines). Our lab did not test these cell lines for mycoplasma contamination.
Commonly misidentified lines<br>(See ICLAC register)	No commonly misidentified cell lines were used in the current study.

Animals and other organisms

Policy information about studies involving animals; ARRIVE guidelines recommended for reporting animal research

Laboratory animals	In this study, two strains of C elegans were used, including N2 and NL5901. The maintenance of the C elegans strains and their treatment with small molecules were carried out by using well established and published protocols. The C elegans strain were mostly female (male population freequency was less than 0.002). The C elegans strains were purchased from CGC (University of Minnesota) and used in the experiments when they were 3 month old.<br>The primary culture neurons were prepared from Pregnant Sprague Dawley Rats (72-85 days old, mixed male and female), which were purchased from Charles River Laboratories (Strain Code 400) and maintained at the University of Denver Animal facility (AAALAC accredited). The rat embryos (both male and female) extracted from Pregnant Sprague Dawley Rats at the embryonic day 18 were used to prepare primary culture neurons. All animal protocols and experiments were approved by the University of Denver Animal Care and Use Committee (IACUC).
Wild animals	No wild animals were used in the study.
Field-collected samples	Fixed brain tissues were obtained from the Carroll A. Campbell, Jr. Neuropathology Lab (CCNL) brain bank at the Medical University of South Carolina (Dr. Steve Carroll, Director) and from Dr. Greg Gerhardt's laboratory at the University of Kentucky. The alpha-synuclein seeds were extracted from the post mortem brain by following a published protocol.
Ethics oversight	No ethical approval required for the C elegans strains. All animal protocols and experiments were approved by the University of Denver Institutional Animal Care and Use Committee (IACUC).

Note that full information on the approval of the study protocol must also be provided in the manuscript.

Flow Cytometry

Plots

Confirm that:

- [x] The axis labels state the marker and fluorochrome used (e.g. CD4-FITC).
- [x] The axis scales are clearly visible. Include numbers along axes only for bottom left plot of group (a 'group' is an analysis of identical markers).
- [x] All plots are contour plots with outliers or pseudocolor plots.
- [x] A numerical value for number of cells or percentage (with statistics) is provided.

Methodology

Sample preparation	Sample preparation details have been provided in the Materials and Methods section and citation from the manufacturer's protocol. Briefly, the HEK293 cells with over expression of WT-Synuclein-YFP or A53T-mutant Synuclein-YFP were used for the experiments. The cells were fixed with paraformaldehyde (4%) and triton-x (0.1%) and then stained with Proteostat dye using the manufacturer's protocol (Proteostat aggregation assay kit).
Instrument	Sony cell sorter (SH800, San Jose, CA) using a 488 nm laser and 525/50 FL2 (YFP) and 600/60 FL3 (Proteostat dye) filters.
Software	Cell Sorter Software (Sony, San Jose, CA).
Cell population abundance	For each experiment, at least 10,000 cells/sample were sorted, counted, analyzed, and plotted and the experiments were conducted in triplicate. However, the data reported in the manuscript is a representation of the three trials.
Gating strategy	At least 10,000 cells/sample were gated to remove any cell debris. A representative Figure has been incorporated in the Supplementary Information that exemplify the gating strategy. The Supplementary Figure no. is Supplementary Fig. 13c.

- [x] Tick this box to confirm that a figure exemplifying the gating strategy is provided in the Supplementary Information.
